# The impact of *SBF2* on taxane-induced peripheral neuropathy

**DOI:** 10.1371/journal.pgen.1009968

**Published:** 2022-01-05

**Authors:** Geneva M. Cunningham, Fei Shen, Xi Wu, Erica L. Cantor, Laura Gardner, Santosh Philips, Guanglong Jiang, Casey L. Bales, Zhiyong Tan, Yunlong Liu, Jun Wan, Jill C. Fehrenbacher, Bryan P. Schneider

**Affiliations:** 1 Department of Medical and Molecular Genetics, Indiana University School of Medicine; Indianapolis, Indiana, United States of America; 2 Department of Hematology and Oncology, Indiana University School of Medicine; Indianapolis, Indiana, United States of America; 3 Department of Clinical Pharmacology, Indiana University School of Medicine; Indianapolis, Indiana, United States of America; 4 Department of Pharmacology and Toxicology, Indiana University School of Medicine; Indianapolis, Indiana, United States of America; University of Minnesota, UNITED STATES

## Abstract

Taxane-induced peripheral neuropathy (TIPN) is a devastating survivorship issue for many cancer patients. In addition to its impact on quality of life, this toxicity may lead to dose reductions or treatment discontinuation, adversely impacting survival outcomes and leading to health disparities in African Americans (AA). Our lab has previously identified deleterious mutations in *SET-Binding Factor 2* (*SBF2*) that significantly associated with severe TIPN in AA patients. Here, we demonstrate the impact of *SBF2* on taxane-induced neuronal damage using an *ex vivo* model of *SBF2* knockdown of induced pluripotent stem cell-derived sensory neurons. Knockdown of *SBF2* exacerbated paclitaxel changes to cell viability and neurite outgrowth while attenuating paclitaxel-induced sodium current inhibition. Our studies identified paclitaxel-induced expression changes specific to mature sensory neurons and revealed candidate genes involved in the exacerbation of paclitaxel-induced phenotypes accompanying *SBF2* knockdown. Overall, these findings provide *ex vivo* support for the impact of *SBF2* on the development of TIPN and shed light on the potential pathways involved.

## Author summary

Taxanes, including paclitaxel, are cancer therapy agents that are widely used with curative potential. However, one of the most common side effects of taxanes, taxane-induced peripheral neuropathy (TIPN), is a devastating survivorship issue for many cancer patients. The employment of taxanes in chemotherapeutic regimens is limited by the severity of TIPN, which may cause lifelong debilitating pain and lack of sensation. Traditionally, African American women have reported higher rates of severe neuropathy with paclitaxel treatment, which has impacted their health outcomes negatively and contributed to treatment disparities. We have previously identified the association of developing severe TIPN with gene *SBF2*, which is a gene known for causing an inherited neuropathy, Charcot-Marie-Tooth disease type 4B2. In this study, we demonstrated that lower expressions of *SBF2* impacted the neuronal morphologic and functional changes resultant from paclitaxel treatment. Moreover, we highlighted candidate genes and inflammatory molecular pathways as potential contributors to the progression of TIPN for future therapeutic target developments.

## Introduction

Taxanes, including paclitaxel, are highly effective antineoplastic agents widely used to treat various solid tumors [1–6]. Although taxanes contribute to improved long-term cancer survival overall, their main toxicity, taxane-induced peripheral neuropathy (TIPN), is one of the most important survivorship issues for patients. The symptoms of TIPN vary between individuals and can be severe and irreversible, negatively impacting routine activities and compromising survivors’ quality of life. For this reason, this toxicity can result in dose reductions or early treatment cessation, thereby impacting patients’ likelihood of relapse [7]. This ultimately contributes to disparities seen in populations that are at a higher risk of developing severe TIPN. Specifically, African American (AA) patients have been found to have markedly higher rates of moderate and severe neuropathy [8].

To date, there are no known effective treatments to prevent or treat TIPN. A lack of drug targets highlights the importance of understanding the impact and corresponding mechanism by which TIPN develops. Prior studies have suggested that TIPN develops, in part, as a result of impaired microtubule dynamicity induced by taxane with subsequent loss of transport function within the axoplasm, affecting primarily peripheral neurons with the longest axons [9–11]. Other studies have suggested that the mitotoxicity of taxanes causes cytoplasmic calcium release resulting in impaired neuronal function [12–17]. The distal generation of reactive oxygen species has also been suggested to contribute to central sensitization, in which the nervous system finds itself in a state of persistent high reactivity [18–20]. While much has been learned, there remain substantial gaps in our understanding of the pathophysiology of TIPN. These gaps in elucidating the mechanism underlying TIPN and identifying novel therapeutic strategies underscore the importance of recognizing which patients are at a higher risk for this toxicity to aid in proper counseling and to consider the risk-to-benefit of treatment.

We previously evaluated germline predictors of TIPN in AA patients with breast cancer, as this population is disproportionately more susceptible to TIPN. Through whole-exome sequencing (WES), we identified the association of five rare variants in *SET-Binding Factor 2* (*SBF2*) with an increased risk of TIPN in AA patients in the randomized phase III adjuvant breast cancer trial, ECOG-ACRIN-5103 [21]. *SBF2* is a biologically plausible candidate gene to investigate in the context of TIPN due to its involvement in a similar pathology, the autosomal recessive polyneuropathy, Charcot-Marie-Tooth Disease Type 4B2 (CMT4B2). In the inherited disease model, it has been suggested that *SBF2* plays a role in regulating phosphoinositide signaling events that contribute to myelination [22], which supports the pathological hallmarks of segmental de- and remyelination exhibited in CMT4B2. Furthermore, it has been demonstrated that *SBF2*’s DENN domain functions as a RAB-specific guanine nucleotide exchange factor [23] and plays a role upstream from autophagosome fusion for vesicle-mediated transport [24].

Of the variants in *SBF2* identified from the WES study, two of them are located in the inactive phosphatase domain that interacts with another protein, MTMR2, for phosphoinositide signaling. The other three variants cluster within proximity of the DENN domain, which may impact autophagosome fusion in membrane trafficking. Hence, the identification of these variants indicating *SBF2*’s dual role in phosphoinositide signaling and vesicle-mediated transport was the basis to investigate the relevance of this gene and all of the potentially understudied pathways involved in the context of TIPN. Although the function of *SBF2* remains incompletely elucidated, identifying its impact on key factors in the development of TIPN has the potential to provide insight to targetable genes or additional pathways in order to ameliorate or reverse TIPN. In this study, we utilize a human-derived *ex vivo* model to assess the impact of *SBF2* on paclitaxel-induced changes in cell viability, morphology, function, and gene expression in human peripheral sensory neurons.

## Materials and methods

### Ethics statement

Approval for the collection of human donor cells was granted by the Indiana University institutional review boards (IRB) 1106005767 and obtained with informed written consent.

### Study design

This study utilized six iPSC lines and siRNA technology to knock down *SBF2* and determine the impact of its decreased expression on the paclitaxel-induced phenotypic changes in donor-derived neurons agnostically to race. We included cell lines from three females of European-descent and three females of African-descent with confirmed *SBF2* wild-type status. Peripheral blood mononuclear cells (PBMCs) were reprogrammed into iPSCs and subsequently differentiated into sensory neurons for assays. iPSC-derived sensory neurons (iPSC-dSN) were cultured for a total of four weeks, at which time siRNA was utilized to knock down *SBF2* and paclitaxel was delivered for a 48-hour treatment. A paclitaxel dose-response experiment was performed to determine a clinically relevant concentration which would detect morphological changes in culture with minimal cell death. The concentration determined (100 nM) was then used for all subsequent assays (viability, morphology, electrophysiology, and RNA sequencing) to compare vehicle- and paclitaxel-treated siRNA negative control (non-targeted control) to vehicle- and paclitaxel-treated si*SBF2* cells. Excluding single-cell sequencing, which compared vehicle- and paclitaxel-treated non-targeted control (NTC) cells from four lines, all experiments described here were assessed in all six independent cell lines.

### Reprogramming PBMCs and generating human iPSC-dSN

PBMCs were obtained with informed written consent collected following Indiana University institutional review boards (IRB) 1106005767 approval and reprogrammed into induced pluripotent stem cells (iPSCs) using the Erythroid Progenitor Reprogramming Kit (STEMCELL Technologies Inc., #05924). Erythroid progenitor cells were isolated and expanded from peripheral blood for 11 days and subsequently reprogrammed into iPSCs with Epi5 Episomal iPSC Reprogramming Kit (Thermo Fisher Scientific, #A15960) vectors via nucleofection. iPSC colonies were obtained within 18–25 days and pluripotency of each line was confirmed by testing the expression of core pluripotency transcription factors from the Human Pluripotent Stem Cell Transcription Factor Analysis Kit (BD Biosciences, #560589) using flow cytometry. Pluripotency was confirmed graphically when the expression pattern of reprogrammed cells was positive for NANOG, OCT3/4, and SOX2, and overlapped with that of an established cell line purchased from WiCell Research Institute, UCSD064i-20-2, which is known to highly express all three transcription factors indicative of pluripotency. The cell cultures were tested for excluding mycoplasma contamination using the MycoAlert Mycoplasma Detection Kit (Lonza, #LT07-118) immediately prior to ensuring a normal karyotype (46,XX) by G-banded cytogenetic analysis, which was performed at the Indiana University Genetic Testing Laboratories. Three cell lines of European ancestry and one of African ancestry were established by these methods for use in the study and confirmed to be wild-type for *SBF2* variants by genotyping. For genotyping, DNA was extracted using the PureLink Genomic DNA Mini Kit (Invitrogen, #K1820-02), and genotyped with TaqMan Genotyping Master Mix (Applied Biosystems, #4371353) and custom TaqMan SNP Genotyping Assays for SNPs rs149501654, rs117957652, rs141368249, rs146987383, and rs7102464 (Assay Ids: C_161562183_10; C_152435684_10; C_161190467_10; C_161447122_10; and C_29019176_10) analyzed by QuantStudio 3 Real-time PCR System Software (Applied Biosystems). Two genetically-confirmed AA iPSC cell lines, STAN245i-601C4 and STAN366i-282C2, were purchased from WiCell Research Institute.

After approximately 20 passages, iPSCs were differentiated into sensory neurons using previously published methods [25]. Briefly, iPSCs were maintained in mTeSR Plus medium (STEMCELL Technologies Inc., #05825) on six-well plates coated with Corning Matrigel GFR Membrane Matrix (Corning, #354230) and with daily media replacement until they reached ~80% confluence. Cultures were then induced into neurogenesis in Dulbecco’s Modified Eagle Medium (DMEM)/F-12 (Thermo Fisher Scientific, #11320033) supplemented with 10% KnockOut Serum Replacement (Gibco, #A31815-01), 0.3 μM LDN-193189 (StemMACS, #130-103-925), 2 μM A83-01 (StemMACS, #130-105-333), 6 μM CHIR99021 (BioVision Inc., #1677–25), 2 μM RO4929097 (Selleck Chemical LLC, #S157510MG), 3 μM SU5402 (Tocris Bioscience, #33–001), and 0.3 μM all-trans-retinoic acid, 97% (ACROS Organics, #207341000), and were maintained in this induction medium through day 8, with media being replaced every 2 days. On day 9, the induction medium was replaced with a maintenance medium that consisted of Neurobasal Plus Medium (Thermo Fisher Scientific, #A3582901) supplemented with 1% B-27 Plus Supplement (Gibco, #A35828-01), 10 ng/mL neurotrophin-3 (Gibco, #PHC7036), 20 ng/mL brain-derived neurotrophic factor (Gibco, #PHC7074), 20 ng/mL nerve growth factor (Gibco, #PHG0126), and 20 ng/mL glial cell line-derived growth factor (Gibco, #PHC7045), with media being replaced every 2 days for 3 weeks. On day 28, iPSC-dSN were positively sorted via magnetic-activated cell sorting (MACS) using anti-PSA-NCAM MicroBeads (Miltenyi Biotec, #130-092-966) and passaged onto a final plate at a seeding density of 3.3 x 10^4^ cells/cm^2^ for subsequent assays.

### Delivery of *SBF2* siRNA and paclitaxel treatment

At day 29, iPSC-dSN received Dharmacon Accell *SBF2* siRNA pool (Thermo Fisher Scientific, #E-014684-00-0020) at 1 μM in Accell siRNA Delivery Media (Thermo Fisher Scientific, #B-005000-500), as specified by the manufacturer. The siRNA pool consisted of 4 target sequences: 5’-ccguuuuccuuagauuauu-3’; 5’-cucuaaagcccaauguaaa-3’; 5’-ccguuauccucaguccauu-3’; and 5’-cccgaguauuuuagaauua-3’. A non-targeting siRNA pool (Thermo Fisher Scientific, #D-001910-10-05) was used as a negative control. Both targeted and non-targeted cells were maintained in delivery media for 72 hours. *SBF2* expression was quantified by RT-qPCR. Total RNA was extracted using the RNeasy Mini Kit (QIAGEN, #74104). The High-Capacity cDNA Reverse Transcription Kit (Thermo Fisher Scientific, #4368814) was used to convert 1 μg of RNA to single-stranded cDNA. RT-qPCR was performed with SYBR Select Master Mix (Applied Biosystems, #4472920) and the following *SBF2* gene-specific primers (Integrated DNA Technologies): forward, 5’-ggaagcaacagcattgtcac-3’; reverse, 5’-tctgaatcttcaaacccactctc-3’. *SBF2* mRNA levels in si*SBF2* cells were normalized to NTC cells and GAPDH was used as an endogenous control. Relative Quantification was determined by the comparative Ct (ΔΔCt) method. On day 32, cells were treated for 48 hours with either vehicle (1% DMSO) or paclitaxel (Tocris Bioscience, #1097) at 100 nM, 300 nM, or 1000 nM diluted in neural maintenance medium.

### Cell viability

Viability was assessed 120 hours post-siRNA delivery (Day 34) by measuring ATP using the CellTiter-Glo One Solution Assay (Promega, #G8461). MACS-sorted cells were treated with paclitaxel 48 hours prior to the viability assessment. Luminescence readings were expressed in relative light units (RLU), which are directly proportional to the amount of ATP present. Readings were normalized to the vehicle-treated NTC cells. Statistical analyses were performed using the results of six independent experiments with four technical replicates for each sample and are described in the statistical analyses section.

### High-content imaging and quantification of neurite outgrowth

On day 34, iPSC-dSN treated with either vehicle or paclitaxel were stained for immunofluorescence. iPSC-dSN were fixed with 4% paraformaldehyde and nuclei were stained with NucBlue Fixed Cell ReadyProbes Reagent (1:1000, Invitrogen, #R37606). Neurites were stained for peripheral neuron marker βIII-tubulin (1:2000, Abcam, #ab41489) and imaged at 4X magnification using the Lionheart FX Automated Microscope (BioTek Instruments, Inc.). A central field of 2017 x 1489 μm from deconvoluted stitched TIFF images in each cell culture well was acquired and automated neurite outgrowth analyses were performed using the Neurite Outgrowth Module in the MetaMorph Microscopy Automation and Image Analysis Software (Molecular Devices). At least 1000 cells were imaged for each condition. Measurements from six independent cell lines were compiled to account for genomic variability.

### Electrophysiology

iPSC-dSN were seeded at 3.95 x 10^4^ cells/cm^2^ on 5 x 5 mm plastic coverslips coated with matrigel. Cells were cultured and maintained on coverslips for the first 48 hours followed by siRNA pool delivery for 72 hours and subsequently treated with either vehicle or paclitaxel for 48 hours, as described above. Whole-cell patch-clamp recordings were conducted as previously reported [26, 27] in voltage-clamp mode at room temperature (∼22°C) using Axopatch 200B patch-clamp amplifier (Molecular Devices). Data were acquired on a Windows-based Pentium IV computer using the pClamp (8.0) software (Molecular Devices). Fire-polished electrodes (1.5–2.5 MΩ) were fabricated from borosilicate glass capillaries (Sutter Instrument Company) using a P-97 puller (Sutter Instrument Company). The standard electrode solution consisted of 140 mm CsF, 10 mm NaCl, 1.1 mm EGTA, and 10 mm HEPES, pH 7.3. The standard extracellular bathing solution contained 130 mm NaCl, 30 mm TEA chloride, 1 mm MgCl2, 3 mm KCl, 1 mm CaCl2, 0.05 mm CdCl2, 10 mm HEPES, and 10 mm D-glucose, pH 7.3.

### Single-cell RNA-sequencing (scRNA-seq) analysis

Cells were dissociated with Accutase (Innovative Cell Technologies Inc., #AT104-500) and were counted with a hemocytometer to assess the number and viability of cells suspended in DMEM/F-12 with 2% bovine serum albumin (BSA). Single-cell gene expression analysis was conducted using a 10X Chromium single-cell system (10X Genomics, Inc) and a NovaSeq 6000 sequencer (Illumina, Inc). A recovery of 1,000–10,000 cells per sample was targeted in single-cell master mix with lysis buffer and reverse transcription reagents, following the Chromium Single Cell 3’ Reagent Kits V3 User Guide, CG000183 Rev A (10X Genomics, Inc). Along with the single-cell gel beads and partitioning oil, the single-cell master mixture containing the single-cell suspension was dispensed onto a Single Cell Chip B in separate wells. The chip was loaded onto the Chromium Controller for GEM generation and barcoding, followed by cDNA synthesis and library preparation. At each step, the quality of cDNA and library was examined by a Bioanalyzer and Qubit equipment. The resulting library was sequenced in a custom program for 28b plus 91b paired-end sequencing on Illumina NovaSeq 6000. A Phred quality score (Q score) was used to measure the quality of sequencing. About 50K reads per cell were generated and 91% of the sequencing reads reached Q30 (99.9% base call accuracy). The analysis was carried out in R (3.6.2) using Seurat (3.1.2) [28], DoubletFinder (2.0.3) [29] and edgeR (3.28.1) [30]. The individual datasets from paclitaxel- and vehicle-treated cell lines were preprocessed to remove low-quality cells based on gene count and mitochondrial content. The ‘sctransform’ function was utilized to normalize and identify features that exhibited high variability across cells. Principal component analysis (PCA) was performed, followed by the identification of the nearest neighbors and generation of cell clusters. The R package, DoubletFinder, was utilized to identify and remove doublets within each individual dataset. The individual datasets were further normalized and variable features were identified. Datasets were then integrated by identifying integration anchors that represent a shared biological state across them. PCA, identification of nearest neighbors, and cluster generation were repeated on the integrated dataset. UMAP dimensional reduction was performed to obtain a two-dimensional representation of the cell clusters. To reduce potential discrepancies that may arise from automated database annotation, meticulous literature search was conducted to select widely recognized genetic markers of mature sensory neurons [31–34]. Clusters showing high expression of marker genes for mature sensory neurons and the absence of proliferative markers were extracted. The sum of the counts for each gene across the cells within a dataset was calculated and genes with read counts per million (CPM) > 0.5 in at least 2 or more datasets were retained. The likelihood ratio test was used to perform the differential analysis between paclitaxel- and vehicle-treated datasets in edgeR. Differentially expressed genes with FDR < 0.05 (Benjamini-Hochberg corrected) were considered significant.

### RNA-sequencing and data analysis

Four weeks after induction, total RNA was isolated from NTC and si*SBF2* cells treated with vehicle or 100 nM paclitaxel using the RNeasy Mini Kit (QIAGEN, #74104). Total RNA was first evaluated for quantity and quality using the Agilent Bioanalyzer 2100. All RNA used had a RIN number of 7 or higher. 100 ng of total RNA was used for each sample. cDNA library preparation included mRNA purification/enrichment, RNA fragmentation, cDNA synthesis, ligation of index adaptors, and amplification, following the KAPA mRNA Hyper Prep Kit Technical Data Sheet, KR1352 –v4.17 (Roche Corporate). Each resulting indexed library was quantified and its quality assessed by a Qubit and Agilent Bioanalyzer; multiple libraries were pooled in equal molarity. The pooled libraries were then denatured and neutralized before loading onto the NovaSeq 6000 sequencer at a final concentration of 300 pM for 100 base paired-end sequencing (Illumina, Inc.). Approximately 30M reads per library were generated. A Q score was used to measure the quality of sequencing; more than 90% of the sequencing reads reached Q30 (99.9% base call accuracy). The sequencing data was assessed using FastQC (Babraham Bioinformatics) for quality control. All sequenced libraries were mapped to the human genome (hg19) using STAR RNA-seq aligner with the following parameter: “—outSAMmapqUnique 60”. The reads distribution across the genome was assessed using bamutils (from ngsutils). Uniquely-mapped sequencing reads were assigned to hg19 refGene genes using featureCounts (from subread) with the following parameters: “-s 2 -p -Q 10”. Quality control of sequencing and mapping results were summarized using MultiQC. Genes with CPM > 0.5 in at least 6 or more datasets were retained. The data was normalized using the trimmed mean of M values (TMM) method. Differential expression analysis was performed using the quasi-likelihood pipeline in the EdgeR (3.28.1) package in R (3.6.2). The variancePartition (1.16.1) was used to assess the interaction between si*SBF2* and paclitaxel treatment. Differential expression with FDR < 0.05 (Benjamini-Hochberg corrected) was considered significant.

### Gene set enrichment analysis

Gene set enrichment analysis (GSEA) was performed following the GSEA guideline (The Broad Institute). The normalized gene expression counts were used to query the following the Hallmark gene set to identify significantly enriched pathways. Gene sets were permutated 1000 times to obtain empirical FDR corrected p-values. An FDR with corrected q-value < 0.25 was used as the cutoff of statistical significance.

### Statistical analyses

The comparison of mean values for continuous variables was conducted with a t-test. A one-way ANOVA was used to test significant differences in viability and average neurite outgrowth changes among groups of iPSC-dSN of various drug concentrations. A mixed two-way ANOVA was used for paired samples to determine significant changes in cell viability and average neurite outgrowth changes between vehicle- and paclitaxel-treated NTC and *SBF2* knockdown (si*SBF2*) cells by using the rstatix package in R (3.6.2). Raw values were used to conduct statistical tests and the relative values were plotted on bar graphs. A two-way ANOVA was used by pairs to identify significant differences in current densities, amplitude, membrane capacitance, and soma size between vehicle- and paclitaxel-treated NTC and si*SBF2* cells. Differential gene expression analysis for RNA-seq data was conducted in the edgeR package with a negative-binomial model. A linear mixed model for repeated measures using the Dream method was adopted to test for significance of interaction. The statistical analyses and data visualization were conducted in R (3.6.2). A p-value less than 0.05 was considered statistically significant. In cases where multiple testing applied, FDR was used and the corrected q-value < 0.05 was used as the cutoff of statistical significance.

## Results

### Paclitaxel decreases average neurite outgrowth but not viability of iPSC-dSN

**Fig 1:**
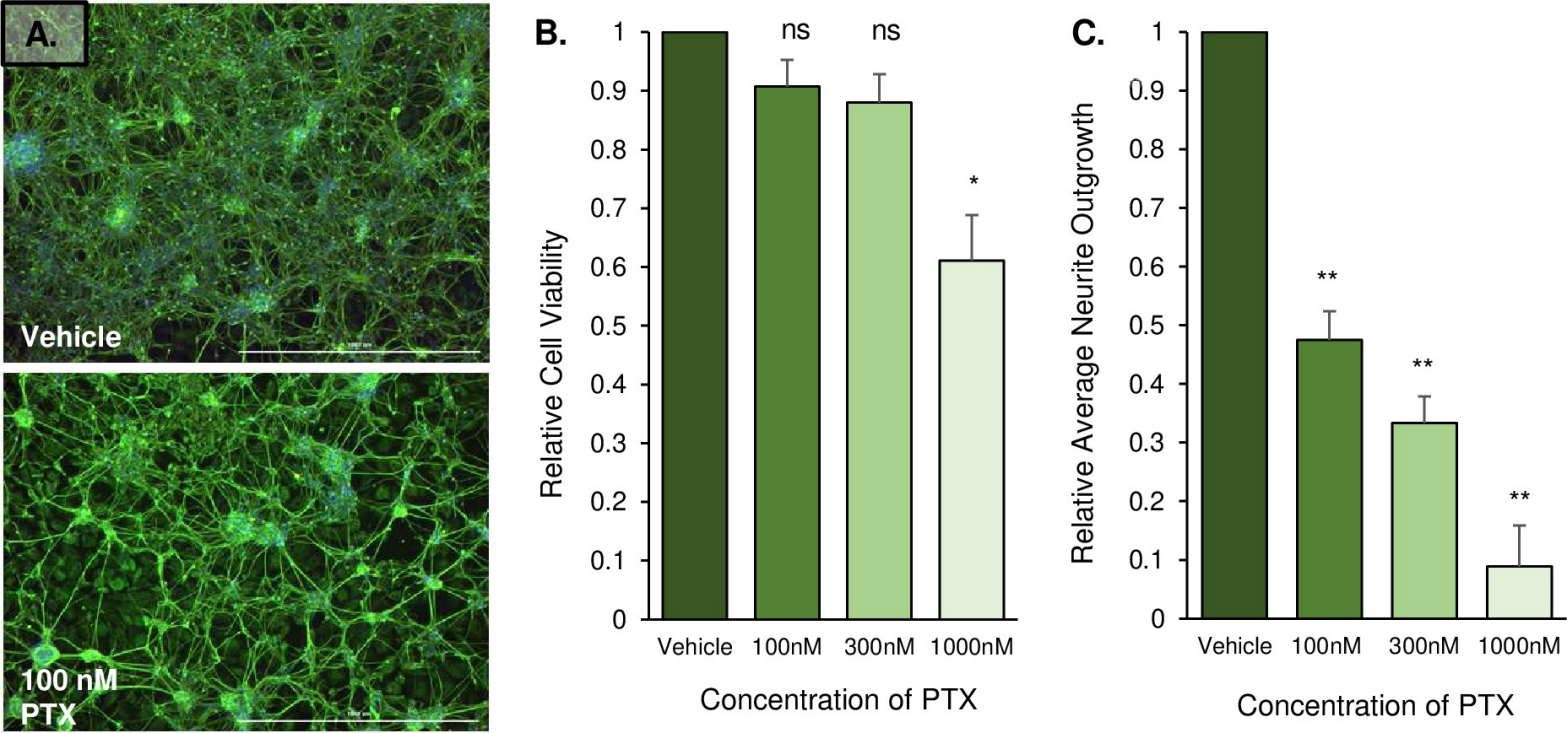
Paclitaxel decreases average neurite outgrowth but not viability of iPSC-dSN. A) Representative immunofluorescent images of iPSC-dSN treated with vehicle or 100 nM paclitaxel for 48 hours. Neurite outgrowths are stained by βIII-tubulin (green) and cell nuclei are stained with Hoeschst 33342 dye (blue). Scale bars = 1000 μm. B) Viability of paclitaxel-treated iPSC-dSN relative to vehicle treatment. C) Average neurite outgrowth of paclitaxel-treated iPSC-dSN relative to vehicle treatment. Each experiment was conducted with technical triplicates for each of the six cell lines. (PTX = paclitaxel, ns = non-significant, * = p<0.05; ** = p<0.01). Error bars represent standard error of the mean (SEM).

To assess paclitaxel’s effect on iPSC-dSN, we measured the viability and morphology of cells treated with vehicle (1% DMSO), 100 nM, 300 nM, or 1000 nM of paclitaxel for 48 hours. The range of paclitaxel concentration was based on prior pharmacokinetic studies reporting a plasma concentration range between 80 nM to 280 nM in patients 20 hours after receiving paclitaxel treatment [5, 35–37]. Seeding density and frequency of passaging of iPSC-dSN were optimized within the selected range to achieve reproducible results. A decrease in neuron network density was visually observed over time-lapse (S1 and S2 Videos) and demonstrated by βIII-tubulin stain of iPSC-dSN before and after paclitaxel treatment (Fig 1A). A dose-dependent reduction (Δ) of ATP indicated the decrease of cell viability, but the reduction was only significant at 1000 nM, a concentration that exceeds the clinically relevant range (Δ = -0.39 ± 0.190, p = 0.023) (Fig 1B). Conversely, there was a significant decrease of average neurite outgrowth in a dose-dependent manner within the clinically relevant dose range of 100–300 nM (p = 1.91 x 10^−5^) (Fig 1C). Thus, we conducted the remainder of experiments at the 100 nM paclitaxel concentration, in which there was a significant decrease in average neurite outgrowth in iPSC-dSN (Δ = -0.53 ± 0.070, p = 0.0039), but not in iPSC-dSN viability (Δ = -0.09 ± 0.111, p = 0.90) to best mimic the clinical pathophysiology.

### Silencing *SBF2* in iPSC-dSN exacerbates paclitaxel-induced viability and morphology phenotypes

**Fig 2:**
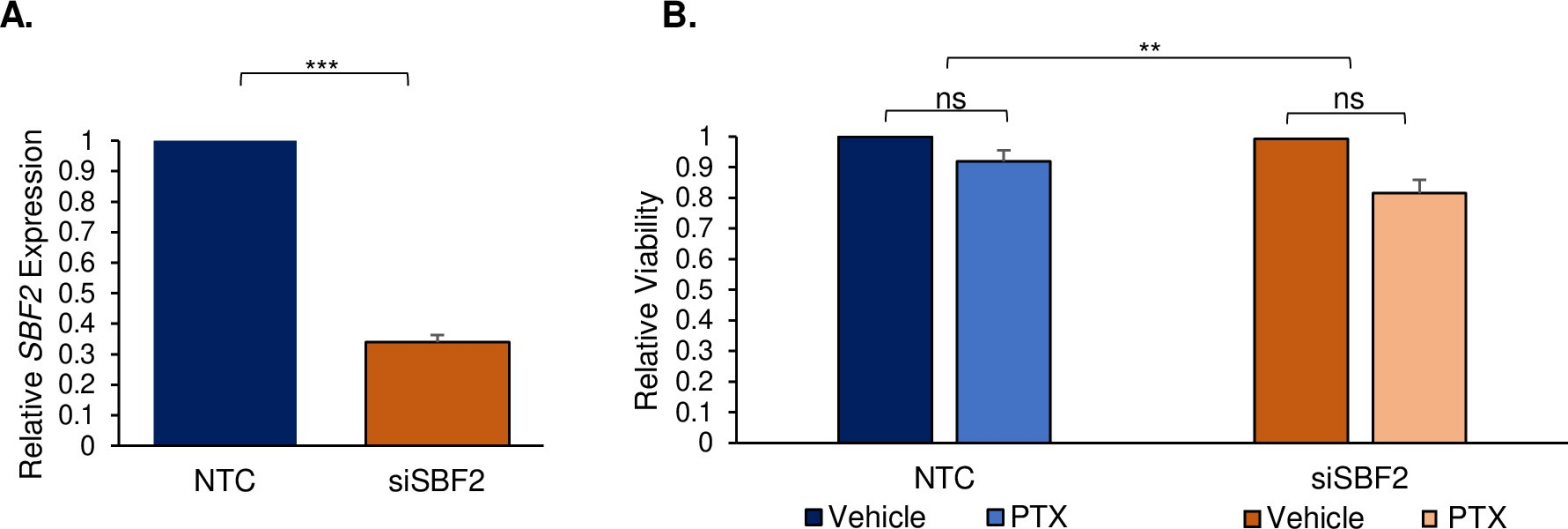
Silencing *SBF2* in iPSC-dSN exacerbates paclitaxel-induced viability phenotype. A) Relative expression of *SBF2* in si*SBF2* cells relative to NTC cells. B) Viability of paclitaxel-treated NTC and si*SBF2* cells relative to baseline (vehicle-treated cells). Graphs represent all six cell lines compiled and performed in triplicates. (NTC = non-targeted control, PTX = paclitaxel, ns = non-significant, ** = p<0.01, *** = p<0.001). Error bars represent SEM.

The impact of *SBF2* knockdown on paclitaxel-induced changes in viability and morphology were evaluated at the concentration of 100 nM. As expected, si*SBF2* cells demonstrated a significant decrease in *SBF2* expression compared to NTC cells (Δ = -0.66 ± 0.056, p = 9.57 x 10^−7^) up to 120 hours following siRNA delivery (Fig 2A). Relative quantification of the average expression of *SBF2* among six cell lines varied by 15.8%.

There was no significant difference in viability between vehicle-treated NTC and si*SBF2* cells at baseline (p = 0.480; Fig 2B). Further, paclitaxel treatment at 100 nM did not significantly decrease viability (p = 0.170) in either the NTC or si*SBF2* cells. The range of average relative cell viability among six cell lines treated with paclitaxel was 81.6–100% (μ = 92% ± 8%). The range of average relative cell viability among si*SBF2* cell lines was 61.7–83% (μ = 81.6% ± 10.5%). However, in evaluating the impact of si*SBF2* across groups, a test for interaction demonstrated a significant exacerbation of decreased viability when comparing the paclitaxel-treated NTC group (Δ = -0.08 ± 0.09) to the paclitaxel-treated si*SBF2* group (Δ = -0.18 ± 0.105) (p = 0.008; Fig 2B).

**Fig 3:**
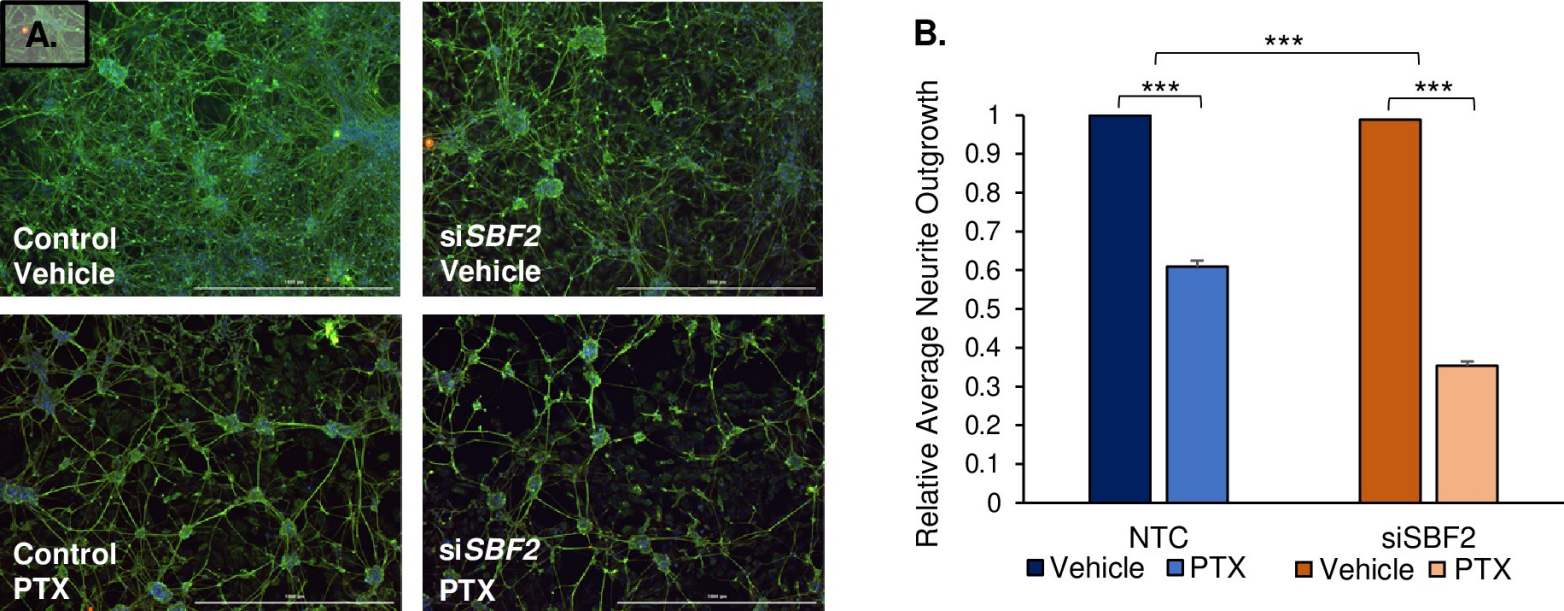
Silencing *SBF2* in iPSC-dSN exacerbates paclitaxel-induced morphology phenotype. A) Representative images of neurite outgrowth. Neurite outgrowths are stained by βIII-tubulin (green) and cell nuclei are stained with Hoeschst 33342 dye (blue). Scale bars = 1000 μm. B) Average neurite outgrowth of paclitaxel-treated NTC and si*SBF2* cells relative to baseline (vehicle-treated cells) for visualization. The graph represents all six cell lines compiled and performed in triplicates. (NTC = non-targeted control, PTX = paclitaxel, ** = p<0.01, *** = p<0.001). Error bars represent SEM.

With respect to morphology, there was an apparent decrease in neuron network density in si*SBF2* cells following paclitaxel treatment (Fig 3A), which was quantified by measuring average neurite outgrowth between NTC and si*SBF2* cells prior to and following paclitaxel treatment. At baseline, there was no significant change to morphology in NTC and si*SBF2* vehicle-treated cells (p = 0.291; Fig 3B). However, there was a significant decrease in both the NTC (Δ = -0.39 ± 0.190) and si*SBF2* groups (Δ = -0.63 ± 0.039) after exposure to 100 nM paclitaxel. The range of average relative neurite outgrowth among six NTC cell lines was 55.6–69.9% (μ = 60.9% ± 4.9%). The range of average relative neurite outgrowth among six si*SBF2* cell lines varied between 35.3–37.6% (μ = 35.8% ± 1.5%). A test for interaction demonstrated that *SBF2* knockdown significantly exacerbated the decrease in average neurite outgrowth induced by paclitaxel (p = 3.03 x 10^−13^; Fig 3B).

### si*SBF2* attenuates paclitaxel-induced sodium currents inhibition

**Fig 4:**
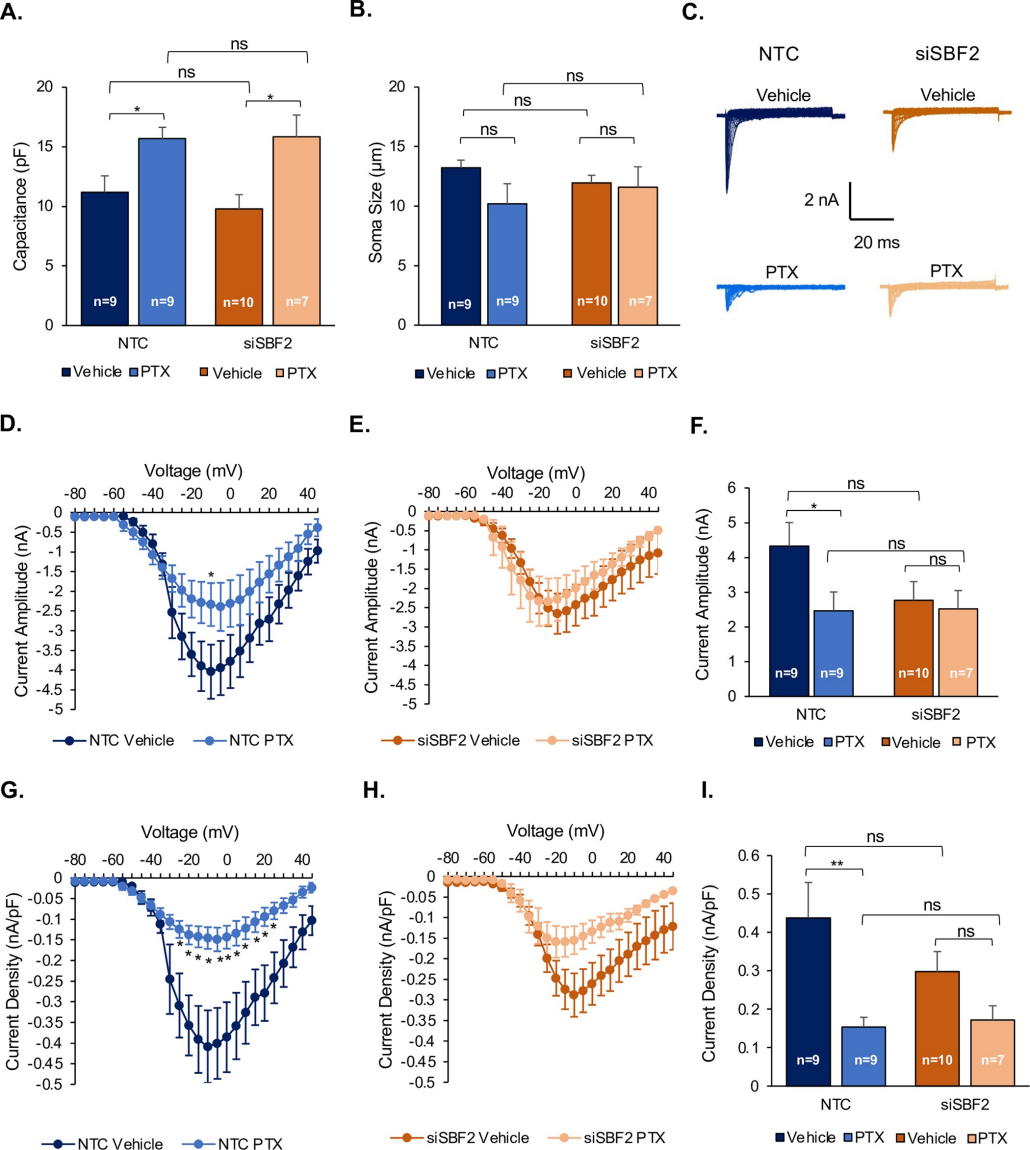
si*SBF2* attenuates paclitaxel-induced sodium currents inhibition. A) Quantification of membrane capacitance. B) Quantification of soma size. C) Representative sodium currents in each group. D) and E) Sodium current amplitude comparing treatment in NTC (D) and si*SBF2* (E) cells. F) Quantification of sodium peak current amplitude. G) and H) Sodium current density comparing treatment in NTC (G) and si*SBF2* (H) cells. I) Quantification of sodium peak current density. Vehicle-treated groups are represented in dark blue (NTC) and dark orange (si*SBF2*); Paclitaxel-treated groups are represented in light blue (NTC) and light orange (si*SBF2*). The number of cells measured for each parameter is represented within each bar. (NTC = non-targeted control, PTX = paclitaxel, ns = non-significant, * = p<0.05, ** = p<0.01). Error bars represent SEM.

To assess functional changes, membrane capacitance and sodium currents of iPSC-dSN were measured. Membrane capacitance determines how rapidly the membrane potential can respond to current changes and therefore influences the excitability of neurons. Knockdown of *SBF2* did not significantly impact capacitance at baseline or after treatment with 100 nM of paclitaxel. However, paclitaxel exposure significantly increased cell capacitance in both the NTC (p = 0.016) and si*SBF2* (p = 0.011) cells (Fig 4A). Since membrane capacitance is proportional to cell surface area, we measured soma size to exclude any contribution to the capacitance changes observed and indeed found no significant differences in any groups tested (Fig 4B). We did, however, observe a difference in the ability to conduct inward sodium currents after paclitaxel treatment in both NTC and si*SBF2* cells, suggesting paclitaxel-induced changes to the excitable properties of cells (Fig 4C). Sodium current density and amplitude were assessed to measure the excitability of neurons with respect to changes in *SBF2* expression and treatment (Fig 4D–4I). While paclitaxel decreased the sodium current amplitude significantly at -10 mV in NTC cells (p = 0.047) (Fig 4D), it did not decrease the amplitude in si*SBF2* cells at any voltage (lowest p = 0.085 at -75 mV, p-values for all the other voltages > 0.3 mV; Fig 4E). Quantification of the amplitude of the peak current demonstrated a significant decrease in amplitude in paclitaxel-treated NTC cells (p = 0.036), but no decrease was observed in paclitaxel-treated si*SBF2* cells (p = 0.077; Fig 4F). Likewise, current density was significantly decreased between -25 and 25 mV in NTC cells treated with paclitaxel (0.01 ≤ p < 0.05; Fig 4G), but no changes were observed in paclitaxel-treated si*SBF2* cells (lowest p = 0.073 at -5 mV; Fig 4H). Quantification of the peak current density demonstrated a significant decrease (p = 0.009) in density in NTC cells, but no decrease was demonstrated in si*SBF2* cells (p = 0.092) following paclitaxel treatment (Fig 4I). At baseline, vehicle-treated NTC and si*SBF2* cells exhibited no significant differences in the density (p = 0.193) and amplitude (p = 0.087) of the peak sodium current at baseline.

### scRNA-seq identifies paclitaxel-induced transcriptomic changes in iPSC-dSN

**Fig 5:**
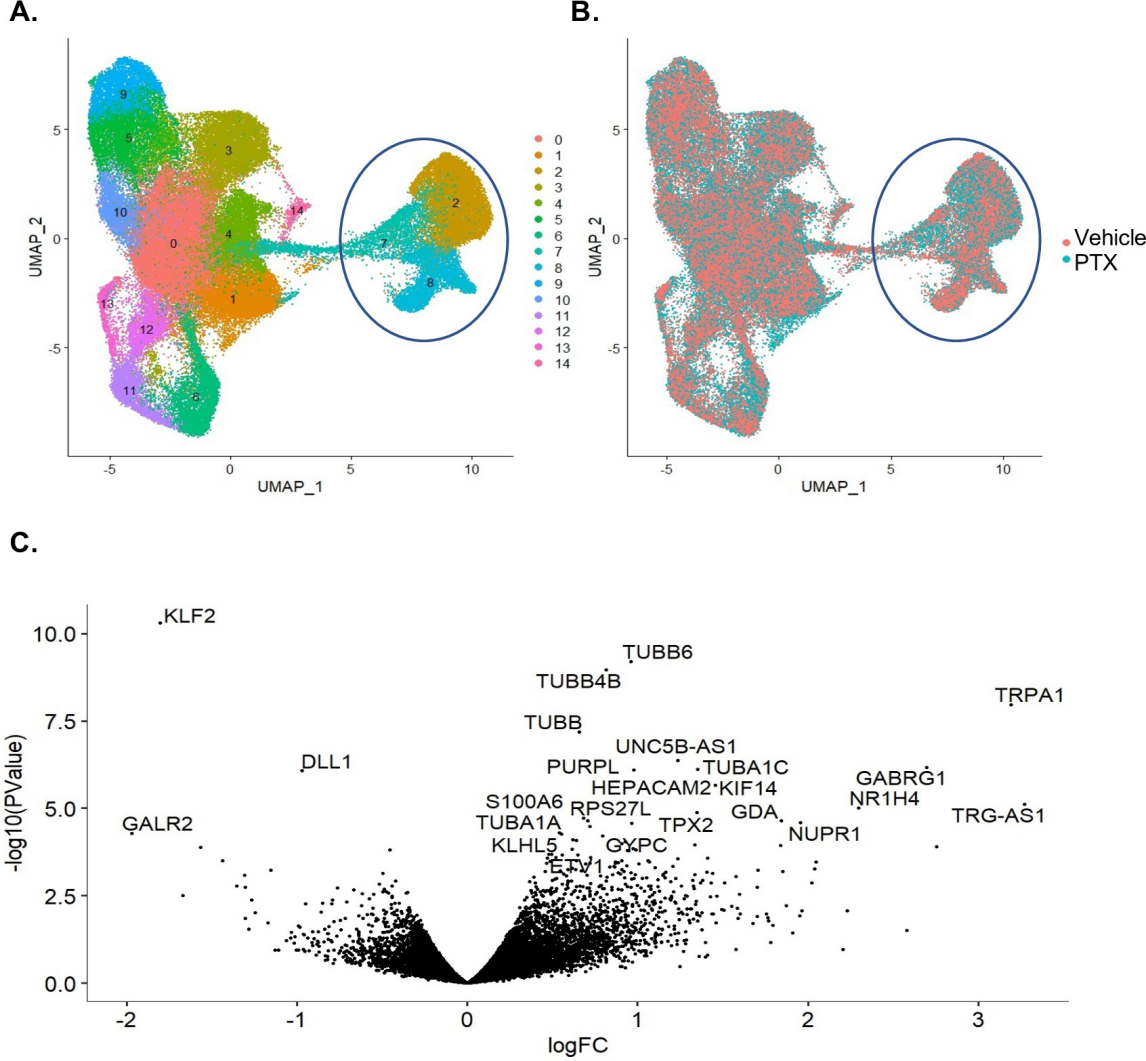
scRNA-seq identifies paclitaxel-induced transcriptomic changes in iPSC-dSN. A) Uniform Manifold Approximation and Projection (UMAP) plot of single-cell transcriptomes. Seurat 3 identified 15 clusters (0–14). B) Overlapping distribution of clustered cells by treatment. Clusters 2, 7, and 8 are characterized as mature sensory neurons and are circled in plots (A) and (B). C) Volcano plot highlighting significant gene expression changes between paclitaxel and vehicle treatment of clusters 2, 7, and 8. A total of 16,601 differentially expressed genes were identified; 21 genes were significantly up-regulated, and 3 genes were significantly down-regulated in the paclitaxel-treated cells (FDR < 0.05). Data obtained from sequencing four iPSC-dSN cell lines. (PTX = paclitaxel, LogFC = log fold-change, FDR = false discovery rate).

With the goal of identifying paclitaxel-induced transcriptomic changes in cultured mature sensory neurons, we first conducted single-cell RNA sequencing (scRNA-seq) for characterizing and profiling individual cells from the iPSC-dSN cultures in addition to confirming the induction of mature sensory neurons in the iPSC-dSN cultures. Cell type-specific clusters (0–14) were identified from the analysis of cells treated with either vehicle or paclitaxel (Fig 5A). Our analysis demonstrates that vehicle- and paclitaxel-treated cells were distributed homogeneously among all clusters (Fig 5B). Mature sensory clusters 2, 7, and 8 were identified and used for differential analyses to compare vehicle- and paclitaxel-treated cells. Paclitaxel exposure at 100 nM concentration impacted the expression of 21 significantly up-regulated and 3 down-regulated genes (Fig 5C). Highly enriched genes that were identified (Table 1) in the analysis are involved in transcription regulation, microtubule dynamics, and neurotransmission processes.

**Table 1:** Differential gene expression following paclitaxel treatment of iPSC-dSN. Comparison between paclitaxel and vehicle treatment of iPSC-dSN in clusters 2, 7, and 8 (FDR<0.05).

Gene	logFC	logCPM	LR	P-value	FDR
TUBB6	0.96	5.41	38.22	6.32E-10	5.25E-06
TUBB4B	0.81	8.28	37.14	1.10E-09	6.08E-04
TRPA1	3.19	1.33	32.70	1.08E-08	4.47E-05
TUBB	0.66	11.20	29.23	6.43E-08	2.13E-04
UNC5B-AS1	1.24	2.98	25.58	4.25E-07	1.18E-03
GABRG1	2.70	-0.61	24.71	6.68E-07	1.41E-03
TUBA1C	1.35	6.31	24.42	7.76E-07	1.41E-03
PURPL	0.98	4.63	24.38	7.92E-07	1.41E-03
KIF14	1.45	0.83	22.44	2.17E-06	3.24E-03
TRG-AS1	3.27	-1.66	20.04	7.59E-06	1.05E-02
NR1H4	2.30	-0.04	19.51	9.98E-06	1.28E-02
HEPACAM2	1.35	3.92	18.96	1.34E-06	1.59E-02
S100A6	0.68	7.21	18.26	1.93E-05	2.13E-02
RPS27L	0.71	8.98	17.98	2.23E-05	2.24E-02
GDA	1.84	-0.50	17.93	2.30E-05	2.24E-02
NUPR1	1.95	0.51	17.72	2.56E-05	2.31E-02
TPX2	0.96	3.24	17.66	2.64E-05	2.31E-02
GYPC	0.72	6.22	17.22	3.33E-05	2.76E-02
TUBA1A	0.54	13.09	16.49	4.90E-05	3.86E-02
KLHL5	0.55	5.88	16.29	5.43E-05	3.92E-02
ETV1	0.79	3.08	16.08	6.09E-05	4.21E-02
KLF2	-1.80	2.61	43.22	4.88E-11	8.10E-07
DLL1	-0.97	4.81	24.25	8.47E-07	1.41E-03
GALR2	-1.97	0.75	16.41	5.11E-05	3.86E-02

Abbreviations used: logFC = log fold-change, logCPM = log counts per million, LR = likelihood ratio, F = F-test, FDR = false discovery rate.

### Paclitaxel-treated si*SBF2* cells reveal candidate genes

**Fig 6:**
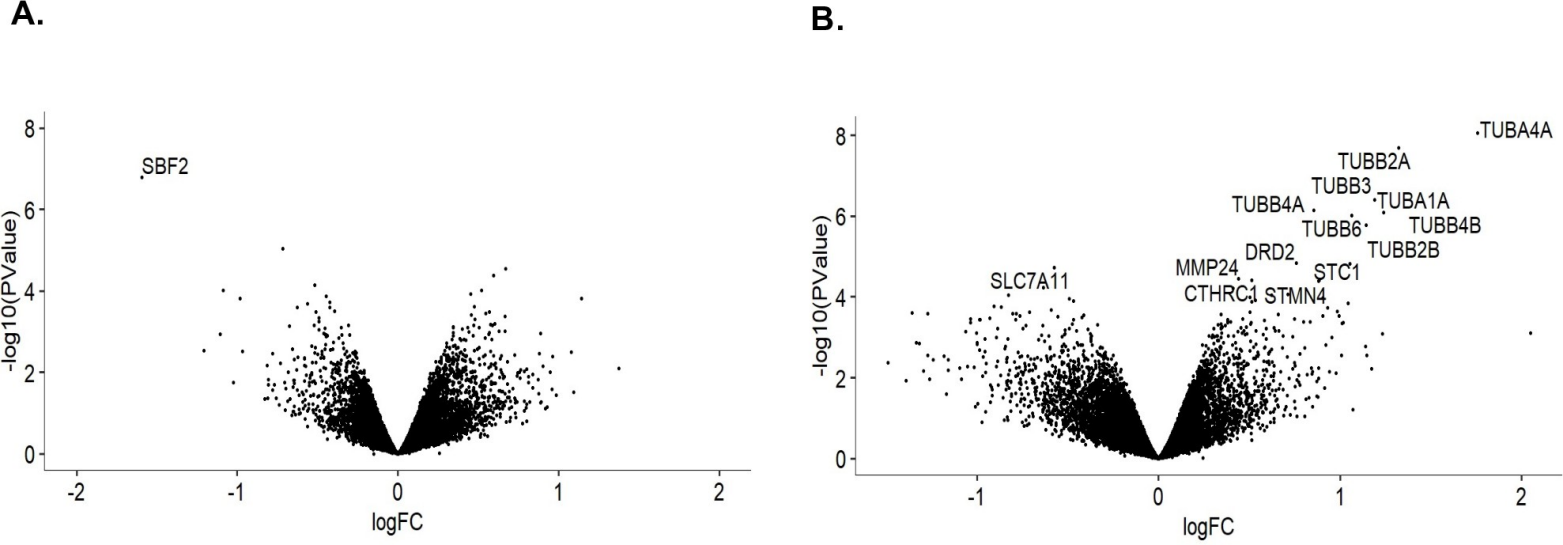
Paclitaxel-treated si*SBF2* cells reveal candidate genes exclusive to this group. A) Volcano plot of the comparison between vehicle-treated NTC and si*SBF2* cells. 15,271 differentially expressed genes were detected in the analysis, with *SBF2* being the only gene that reached statistical significance (FDR = 0.002, logFC = -1.60.) B) Volcano plot of genes differentially expressed between vehicle- and paclitaxel-treated si*SBF2* cells. 15,239 differentially expressed genes were detected, of which 14 reached statistical significance. Data obtained from sequencing six iPSC-dSN cell lines. (PTX = paclitaxel, logFC = log fold-change, FDR = false discovery rate).

With scRNA-seq confirming mature sensory neurons in culture, we set out to analyze the effects of paclitaxel on *SBF2* knockdown cells by comparing transcriptomic profiles from each cell group in pooled RNA-seq. When comparing NTC and si*SBF2* cells at baseline, *SBF2* was the only gene significantly down-regulated in the si*SBF2* cells (logFC = -1.60, p-value = 1.57 x 10^−7^, FDR = 0.0024; Fig 6A), which highlights the specificity of the siRNA pool utilized to silence *SBF2* in the knockdown group. Evaluation of paclitaxel’s effect on expression revealed 13 genes that were significantly up-regulated and 1 gene significantly down-regulated in the si*SBF2* cells after treatment (Fig 6B; Table 2). Among these, an abundance of tubulin genes was up-regulated, similar to the findings in the single-cell work, pointing to changes in microtubule dynamics following paclitaxel treatment.

**Table 2:** Differential gene expression of paclitaxel-treated si*SBF2* iPSC-dSN. Comparison between paclitaxel and vehicle treatment (FDR < 0.05).

Gene	logFC	logCPM	F	p-value	FDR
TUBA4A	1.76	3.16	220.80	8.75E-09	1.33E-04
TUBB2A	1.32	8.54	189.23	2.01E-08	1.53E-04
TUBB3	1.19	9.77	107.34	4.01E-07	2.04E-03
TUBB4A	0.85	6.79	95.60	7.27E-07	2.44E-03
TUBA1A	1.24	11.97	93.64	8.08E-07	2.44E-03
TUBB6	1.06	7.90	90.47	9.62E-07	2.44E-03
TUBB4B	1.14	8.11	81.38	1.64E-06	3.58E-03
DRD2	0.76	4.61	52.26	1.43–05	2.54E-02
TUBB2B	1.06	9.69	51.73	1.50E-05	2.54E-02
SLC7A11	-0.57	6.75	49.47	1.85E-05	2.83E-02
MMP24	0.44	6.17	42.98	3.56E-05	4.32E-02
STC1	0.89	3.23	42.20	3.86E-05	4.32E-02
CTHRC1	0.51	5.95	42.09	3.91E-05	4.32E-02
STMN4	0.88	6.97	41.96	3.97E-05	4.32E-02

Abbreviations used: logFC = log fold-change, logCPM = log counts per million, F = F-test, FDR = false discovery rate.

Further, we identified differential expression of genes that were exclusive to paclitaxel-treated si*SBF2* cells. Of these, *SLC7A11*, an important gene for resistance to oxidative stress was down-regulated, and *MMP24*, which is a gene involved in the process of inflammatory hyperalgesia, was up-regulated in this group. To aid in understanding the processes involving these transcriptomic changes and the development of neuropathy, differentially regulated genes were subjected to Gene Set Enrichment Analysis (GSEA) of hallmark gene sets. Seven gene sets were significant at an FDR< 0.25, with a predominance of these pathways involving inflammatory processes. Three of the enhanced gene sets identified are represented in S1 Fig.

## Discussion

The significant impact of TIPN on cancer patients’ quality of life has mobilized many efforts to improve understanding of the mechanism underlying this toxicity. Diverse approaches have been utilized to identify the direct effects of paclitaxel *in vivo* with rodent behavioral studies [38, 39], and *in vitro* with procured postmortem human dorsal root ganglion tissue [40, 41] and human-derived neurons reprogrammed from iPSCs [42–46], providing a framework to identify general phenotypic features of disease. A major advantage to the use of iPSC-derived neurons is the ability to conduct studies with consideration to a patient’s unique genetic diversity. The study herein is novel in that it builds on our prior work that discovered an association between AA patients with *SBF2* deleterious mutations and a marked increase in risk of clinically significant TIPN. Despite the clear association seen in a large clinical trial, demonstrating the morphological and functional impact of *SBF2* in an *ex vivo* model provides an even higher level of support for the role of *SBF2* with TIPN. Additionally, the comprehensive assessment of the impact of *SBF2* on global gene expression was carried out to uncover critical pathways or genes that might serve as drug targets.

Our viability and morphology studies found that silencing *SBF2* exacerbated the effects of paclitaxel treatment. The effects seen on the si*SBF2* cells at clinically relevant concentrations of paclitaxel mimicked the phenotype of the non-silenced cells when exposed to higher concentrations. These findings parallel a prior study, in which knockdown of tubulin gene *TUBB2A* also resulted in more dramatic changes in total neurite outgrowth following paclitaxel treatment [44] and corroborated prior genomic work that had associated polymorphisms regulating *TUBB2A* expression with patients at a risk of developing TIPN [47]. Altogether, significant change in viability and the dramatic decrease in average neurite outgrowth in paclitaxel-treated si*SBF2* cells in our study support the clinical findings of higher rates of severe grades of neuropathic toxicity at usual doses of the drug in patients with lower levels of *SBF2*.

In the current study, we found that paclitaxel-induced axon degeneration was exacerbated by si*SBF2*, which could suggest that si*SBF2* might decrease sodium channel expression at the distal axons. On the other hand, our study found that paclitaxel decreased sodium currents recorded in the cell soma and si*SBF2* prevented this inhibition. Axon degeneration and alteration in sodium channels are two major mechanisms of paclitaxel-induced TIPN [46]. However, how these two mechanisms interact is still not clear. A recent study suggests paclitaxel-induced axon degeneration is associated with less sodium channel expression at distal axons, and impaired axonal trafficking in primary sensory neurons [46]. Our results suggest that paclitaxel might reduce sensory sensitivity through changes in sodium channels in both soma and distal axons, but si*SBF2* only in distal axons. Thus, si*SBF2* may not increase paclitaxel-induced sensory insensitivity through soma sodium channels as the level of sodium currents is virtually of no difference between si*SBF2* and control vehicle following paclitaxel treatment (Fig 4F–4I). Overall, these results suggest that si*SBF2* might further exacerbate paclitaxel-induced sensory insensitivity by modulating changes to the sodium channels located at distal axons.

The presence of inflammatory mediators has also been shown to affect vesicular trafficking of ion channels, thereby affecting the neuron’s response to stimuli [48–51]. Our transcriptomic data indicated that the inflammatory mediator *MMP24* was significantly up-regulated (Fig 6B) and inflammatory pathways were enriched in paclitaxel-treated si*SBF2* cells (S1 Fig), but not in the paclitaxel-treated control cells. This finding suggests that the inflammatory mechanism may interact with the functional role of *SBF2* on neuron function. Overall, the functional findings discussed here point to paclitaxel and *SBF2*’s functional roles not sharing the same mechanism.

This work also identified key paclitaxel-induced transcriptomic changes specific to mature sensory neurons. Importantly, a number of established microtubule- and neurotransmission-related genes were differentially expressed in the paclitaxel-treated group, which supported the fidelity of the model and subsequent results. Specifically, an abundance of genes from the tubulin family (*TUBB6*, *TUBB4B*, *TUBB*, and *TUBA1C*) were differentially expressed, lending support to the hypothesis that paclitaxel increases the stability of mature tubulin mRNAs in quiescent cells [52]. Other genes impacted included an up-regulated *NUPR1*, which is a known transcriptional regulator that participates in apoptosis, autophagy, and DNA repair responses to microenvironment changes [53, 54]. We subsequently evaluated for the impact of *SBF2* silencing on global expression differences. Here, we again found massive up-regulation of the tubulin family of genes, but also uncovered unique differentially expressed genes.

Our data points to a convergence on inflammation as a mediator of increased risk of taxane-induced neuropathy in the face of low *SBF2* expression. Specifically, GSEA demonstrated the most highly enriched pathways were those critical to immune response; highlighted by interferon-gamma response and tumor necrosis factor-alpha signaling. In the transcriptomic analysis, we demonstrated significant upregulation of *MMP24* which mediates peripheral thermal nociception and inflammatory hyperalgesia [55]. While the mechanism of upregulated *MMP24* in the face of *SBF2* is not completely elucidated, our functional data would support the possibility of causing hypoexcitability through changes in the number of neuronal ion channels on the surface or vesicular trafficking [48–51]. In addition, *SLC7A11* which increases resistance to oxidative stress [56, 57] and in integrated in the inflammatory response pathway, was the only significantly down-regulated gene in paclitaxel-treated si*SBF2* cells. Thus, moving forward, studies targeting candidate genes such as *MMP24* or *SLC7A* or those more globally directed at suppressing inflammation would be rational.

Altogether, these results suggest that *SBF2* may have a functional role in the development of TIPN, potentially by also mediating inflammatory pathways. The phenotypic and functional support here provides further evidence for the development of TIPN predictive biomarker using *SBF2*. Particularly, subgroups of patients in which neuropathy is markedly more common, such as those of African descent, are likely to benefit from identifying the presence of *SBF2* variants preemptively. While the current work does not have adequate power to address the effects of the interaction of paclitaxel-treated *SBF2* knockdown and ancestry, further investigative work disentangling the interaction between race and *SBF2* will be pivotal in our understanding of how race may contribute to the disparities in TIPN. To this end, we are conducting an NCI-sponsored ECOG-ACRIN clinical trial, EAZ171 (NCT04001829), to prospectively test whether breast cancer patients of African descent with germline *SBF2* mutations do indeed have higher rates of TIPN. Additionally, in that study, the collected patient-derived iPSC-dSN will afford the possibility to further evaluate the complex genomic underpinnings of TIPN in the context of race. Ultimately, the use of patient-derived iPSC-dSN as a model allows for the consideration of each patient’s unique genetic diversity and to detect other candidate genes contributing to TIPN. Additional functional studies investigating the role of *SBF2* and of the significant differentially expressed genes identified here will be key to elucidate the underlying biological mechanism for this toxicity. Hopefully these findings will uncover critical pathways for identification of drugs that will ultimately prevent or treat the cadre of side effects, which can be devastating for patients.

## Supporting information

S1 Video Time-lapse of iPSC-dSN treated with vehicle. (MP4)

S2 Video Time-lapse of iPSC-dSN treated with 300 nM paclitaxel. (MP4)

S1 Fig Gene set enrichment analysis of hallmark genes associated with paclitaxel-treated siSBF2 versus control vehicle cells. A) Interferon-gamma response pathway. B) Tumor necrosis factor–alpha signaling via nuclear factor kappa-light-chain-enhancer of activated B cells pathway. C) Inflammatory response. Normalized enrichment scores (NES) are highlighted below, and an FDR<0.25 was significant.
(TIFF)
