# Supplementary figures and images for: The impact of SBF2 on taxane-induced peripheral neuropathy
Source: PLoS Genet. 2022 Jan 5;18(1):e1009968. doi: 10.1371/journal.pgen.1009968 (PMC8765656; doi:10.1371/journal.pgen.1009968)

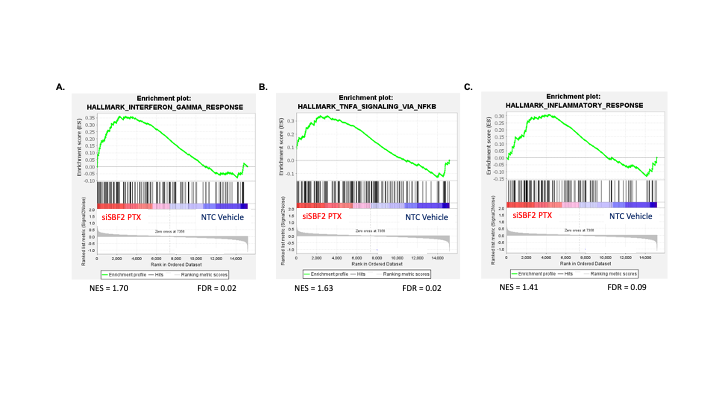

Supplement: S1 Fig — A) Interferon-gamma response pathway. B) Tumor necrosis factor–alpha signaling via nuclear factor kappa-light-chain-enhancer of activated B cells pathway. C) Inflammatory response. Normalized enrichment scores (NES) are highlighted below, and an FDR<0.25 was significant. (TIFF) [file pgen.1009968.s003.tiff]
